# Differences in resilience, hope, and sense of coherence among adolescents with ADHD/DCD, co-occurring ADHD and DCD, and typically developing adolescents

**DOI:** 10.1371/journal.pone.0357815

**Published:** 2026-09-15

**Authors:** Yael Rozman, Miri Tal-Saban

**Affiliations:** School of Occupational Therapy, Hebrew University, Jerusalem, Israel; University of Oviedo, SPAIN

## Abstract

**Purpose:**

Adolescents with attention-deficit/hyperactivity disorder (ADHD) and developmental coordination disorder (DCD) face daily challenges, which intensify when both disorders co-occur. Resilience, hope, and sense of coherence (SOC) support positive adaptation, yet research in this area is limited. This study examined resilience, hope, and SOC among adolescents with ADHD/DCD, co-occurring ADHD and DCD, and typical adolescents.

**Methods:**

105 adolescents (12–18 years) with ADHD/DCD (n = 37), co-occurring ADHD+DCD (n = 23), and typical adolescents (n = 45) underwent a comprehensive assessment to confirm their diagnostic status before completing self-report questionnaires on resilience, hope, and SOC.

**Results:**

The ADHD+DCD group reported significantly lower levels of resilience (*p* = .017), hope (*p* = .001), and SOC (*p* = .007) compared to typical adolescents. No significant differences were found between the ADHD+DCD and the ADHD/DCD groups in all the main study variables (*p* > .05). Moderate to strong positive correlations were found linking resilience, hope, and SOC, with hope and SOC accounting for the majority of resilience variance and the final model explaining 54.7% of the variance.

**Conclusions:**

These preliminary findings suggest that adolescents with co-occurring ADHD and DCD may experience lower resilience, hope, and SOC than TD peers. These findings highlight the importance of considering co-occurring ADHD and DCD as a clinically relevant presentation and addressing adolescents’ emotional and adaptive resources within assessment and intervention.

## Introduction

Adolescence, as defined by the World Health Organization (WHO), is the transitional phase between childhood and adulthood, spanning the ages of 10–19 [1]. This period is characterized by rapid physical, emotional, and social changes, often accompanied by stressful and unforeseen life events [2]. The ability to recover and adapt to these challenges is referred to as resilience [3,4]. Resilience enables individuals to respond adaptively to adversity and stress while focusing on their strengths [5,6]. Resilience is influenced by the interplay of biological, psychological, social, and cultural factors [7]. Individuals with higher resilience levels often exhibit curiosity and openness to new experiences, facilitating positive coping mechanisms in the face of uncertainty, conflict, and failure [8,9].

Additional constructs contributing to resilience include hope [10–12] and sense of coherence (SOC) [13]. Hope is defined as a cognitive framework that reflects an individual’s perceived ability to identify pathways to achieve personal goals and maintain motivation despite adversities [14]. Hope is a forward-looking, realistic, and multifaceted construct that serves as a vital resource for health and health-promoting processes [15]. High levels of hope positively influence mood and functioning, serving as a protective factor against challenging experiences [10,16]. SOC is a concept proposed by Antonovsky [17] and defined as a global orientation toward life, which refers to an internal perception that the world is comprehensible, manageable, and meaningful. Individuals with a strong SOC are better equipped to cope with stress and maintain good health [17,18]. Both hope and SOC have been shown to positively influence fundamental aspects of health [19].

Adolescents with neurodevelopmental disorders (NDDs) face unique daily challenges, including significant difficulties in participating in everyday activities and managing social, emotional, and behavioral issues [20,21]. Among the prevalent NDDs are attention-deficit/hyperactivity disorder (ADHD), with an estimated global prevalence of 8% among children and adolescents [22] and developmental coordination disorder (DCD) which affects approximately 5% of children and adolescents [23], with associated difficulties persisting into adulthood in up to 70% of individuals [24].

According to the Diagnostic and Statistical Manual of Mental Disorders, 5th edition – Text Revision (DSM-5-TR) [25], ADHD is characterized by persistent patterns of inattention, hyperactivity, and impulsivity. Adolescents with ADHD often exhibit lower resilience, which contributes to academic difficulties, low self-esteem, anxiety, and depression [26,27].Adolescents with ADHD have been found to report lower resilience than their unaffected siblings and Typically Developing (TD) peers [27], while an integrative review linked lower resilience in ADHD with adverse academic and emotional outcomes [26]. Evidence concerning SOC is more preliminary. Stronger SOC has been associated with greater life satisfaction among adolescents with ADHD symptoms [28] and with fewer ADHD symptoms over time [29]. However, these studies did not establish that adolescents with ADHD have lower SOC than TD peers, and empirical evidence regarding hope in this population remains scarce.

According to the DSM-5-TR, DCD is characterized by persistent difficulties in acquiring and executing motor skills, which manifest as clumsiness, slowness, and inaccuracy in movement, significantly impacting daily functioning [25]. Available evidence concerning positive adaptation resources in DCD is limited and developmentally fragmented. Adults with diagnosed or suspected DCD have reported lower resilience and self-efficacy and higher anxiety than TD adults, with greater resilience associated with lower anxiety [30]. Among young children, those with DCD reported lower hope and SOC than children without DCD [31]. Although these findings suggest that positive adaptation resources may be relevant across the lifespan, differences in age, samples, and constructs limit their generalizability. In particular, it remains unclear whether similar patterns are present among adolescents with DCD.

A substantial number of individuals with DCD are also diagnosed with ADHD, and vice versa, with co-occurrence rates reported to be as high as 50% [32]. Co-occurrence refers to the simultaneous presence of two disorders, which may result in unique challenges that are not accounted for by examining each condition independently [33]. Emerging evidence suggests that adolescents with both ADHD and DCD display distinct neurobiological patterns and more severe symptoms, compared to those with either condition alone [34]. This population often exhibits greater motor, cognitive, and social challenges, emphasizing the importance of understanding their resilience profiles and related constructs [35]. However, direct comparisons between adolescents with co-occurring ADHD+DCD and those with a single diagnosis remain limited [34].

Resilience, hope, and SOC are critical constructs that may support adolescents in coping with the challenges associated with NDDs [10,36–39]. Nevertheless, the available literature has generally examined these constructs separately and across different developmental stages [27–31]. Their combined examination among adolescents with ADHD/DCD, and ADHD+DCD, compared with TD peers, therefore remains limited.

Our study aimed to address this gap by exploring differences in resilience, hope, and SOC among groups with single diagnosis ADHD/DCD, co-occurring ADHD+DCD, and TD. By examining these constructs, the findings may inform targeted interventions to enhance the well-being of adolescents with NDDs [31,40]. Furthermore, a better understanding of resilience, hope, and SOC across these groups may inform individualized assessment and support; however, further research is needed to establish their implications for intervention. Therefore, further research in this area is crucial [30,38,41,42].

To our knowledge, this preliminary study is the first to compare populations with ADHD/DCD, and their co-occurrence, in an attempt to identify their effects on resilience, hope, and SOC. Our specific aims were to: (1) assess differences in resilience, hope, and SOC among adolescents with a sole diagnosis of ADHD or DCD, those with co-occurring diagnoses, and their TD peers; and (2) explore the contributions of diagnosis status (sole diagnosis, or co-occurring diagnoses) and the constructs of hope and SOC to resilience variance.

### Hypotheses

Based on previous findings, we hypothesized that adolescents with single diagnosis of ADHD/DCD would report lower levels of resilience, hope, and SOC compared to TD peers [26,28,30]. Due to the limited research comparing co-occurring ADHD and DCD to single diagnoses and given evidence of more severe symptoms in co-occurring presentations [34,43], we further expected the co-occurrence group to report lower levels of these constructs than the other groups. Finally, we hypothesized that hope, SOC, and resilience would be positively interrelated, reflecting mutual associations among these constructs [10–13,44].

## Methods

### Participants

Convenience sampling was used via social media to recruit study participants. A total of 177 adolescents and their guardians responded to the study advertisements. Inclusion in the study groups followed the DSM-5-TR [25] criteria for ADHD and DCD. Adolescents were included in the ADHD group if their guardians reported in the demographic questionnaire that they had previously received an ADHD diagnosis from a physician and if at least six of the nine items in either the Inattention or Hyperactivity-Impulsivity subscale of the ADHD rating scale questionnaire (ADHD-RS) were rated 2 (“often”) or 3 (“very often”) [45].

Those included in the DCD group met the following: (a) previous physician diagnoses of DCD were reported by the participants’ guardians in the demographic questionnaire, or they scored  < 58 on the Developmental Coordination Disorder Questionnaire (DCDQ) [46], or > 26 on the Adolescents and the Adults Coordination Questionnaire (AAC-Q) [24], based on their age; and (b) They scored below the 16^th^ percentile on the Movement Assessment Battery for Children – second edition (MABC-2) [47], which was administered by occupational therapists; and (c) They scored normally on the ADHD-RS [45] questionnaire. Adolescents who met both ADHD and DCD criteria were classified as having co-occurring ADHD+DCD.

Of the 177 who responded to the recruitment efforts, 42 adolescents withdrew during the eligibility screening process, 13 were excluded due to reports of significant health conditions other than ADHD or DCD, and 14 adolescents reported as TD were excluded for scoring above the cut-off for ADHD or DCD on the screening measures. Additionally, three adolescents with DCD were excluded because their M-ABC-2 scores exceeded the 15th percentile.

The remaining 105 adolescents were aged 12–18 (mean age = 15.23, SD = 1.79, 50.5% males and 48.5% females, 1% other). These were categorized into three groups: (a) 37 adolescents with ADHD/DCD (mean age = 14.96, SD = 1.87, 67.6% males); (b) 23 adolescents with co-occurring ADHD+DCD (mean age = 15.66, SD = 1.45, 43.5% males); and (c) 45 TD adolescents (mean age = 15.24, SD = 1.86, 40% males, 57.8% females, 2.2% other). See Table 1.

**Table 1:** Demographic characteristics of the study participants.

		ADHD/DCD	ADHD+DCD	TD
		*n* = *37*	*n* = 23	*n* = 45
		n (%)	n (%)	n (%)
**Sex**	Male	25 (67.6)	10 (43.5)	18 (40.0)
	Female	12 (32.4)	13 (56.5)	26 (57.8)
	Other			1 (2.2)
**Living area**	Urban	12 (32.4)	14 (60.9)	20 (44.4)
	Rural	25 (67.6)	9 (39.1)	25 (55.6)

Participants in all groups were excluded if guardians reported a diagnosed psychiatric condition other than ADHD or DCD, including anxiety or depressive disorders, or any significant medical, neurological, physical, or sensory condition. Participants in all groups attended regular education settings and did not use medications other than those prescribed for ADHD. Among participants in the ADHD/DCD group, 60.7% used medication, and in the co-occurrence group, 60.9% used medication.

In the absence of research addressing differences in resilience among these populations, sample size was determined using G*Power software [48], based on the study of Dewey and Volkovinskaia [49]. Based on an anticipated effect size of f^2^ = 0.149, 90% power, and a type-I error of 0.05, it was calculated that a total of 56 participants would be needed.

### Screening measures

#### Demographic questionnaire.

A parent-report questionnaire developed for the current research, to gather demographic details such as age, gender, educational background, living environment, religion, etc*.* The demographic questionnaire also included questions regarding previously diagnosed neurodevelopmental, psychiatric, neurological, or medical conditions.

#### ADHD Rating Scale IV (ADHD RS IV)[45].

A parent-report screening questionnaire measuring ADHD symptoms according to the Diagnostic and Statistical Manual of Mental Disorders (DSM-5) criteria designed for children and adolescents aged 5–18 suspected of having ADHD. Parents rate 18 items representing their child’s frequency of ADHD symptoms in the past six months on a scale from 0 (“not at all”) to 3 (“a lot”). A child has suspected ADHD when at least six out of the first nine items, or nine out of the total items, receive a score of 2 or 3. The questionnaire has been found to be valid and reliable, demonstrating good psychometric properties [45,50]. The ADHD-RS was used as a symptom-specific measure to corroborate the presence of current core ADHD symptoms for group-classification purposes. ADHD status was not determined based on the ADHD-RS alone, in accordance with existing literature [50,51].

### The Developmental Coordination Disorder Questionnaire-2007 (DCDQ’07)[46]

This parent-report screening questionnaire is designed to measure DCD symptoms in children aged 5–15. Parents assess 15 items that reflect their child’s motor performance, compared to peers, using a scale from 1 (“does not describe my child at all”) to 5 (“describes my child perfectly”). The total score on the questionnaire is the sum of scores across all items, ranging from 15 to 75. A score below 58 suggests suspected DCD [46]. The questionnaire has been found to be valid and reliable, demonstrating good psychometric properties [46,52,53].

### The Adolescents and Adults Coordination Questionnaire (AAC-Q)[24]

This self-report screening questionnaire is designed to identify DCD among adolescents and adults aged 16–30 years. Adolescents rate 12 items representing current and childhood difficulties in motor-related daily functions based on their occurrence, using a scale ranging from 1 (“never or 0% of the time”) to 5 meaning (“always or 100% of the time”). The total score on the questionnaire is the sum of scores across all items, ranging from 12 to 60. A score > 26 (i.e., 0–15 percentile) indicates borderline or probable DCD. The questionnaire has been found to be valid and reliable, demonstrating good psychometric properties [24].

### The Movement Assessment Battery for Children, second edition (MABC-2)[47]

This is a standardized diagnostic tool for assessing DCD, which evaluates both gross- and fine-motor skills among children aged 3–16 years. It includes eight tasks across three domains: manual dexterity, aiming and catching, and balance. In the current study, the cutoff point for the DCD group/co-occurring group was set at the 16th percentile. The MABC-2 has been found to be valid and reliable, demonstrating good psychometric properties [47,54].

MABC-2 considered a gold standard for diagnosing DCD [32]. At the time of data collection, no standardized motor assessment was available for individuals older than 16 years. Therefore, consistent with previous studies [55–57] and according to evidence supporting its feasibility for assessing motor performance and its use as an inclusion criterion in adult samples, the MABC-2 was also administered to participants aged 17–18 years [58,59]. In the current study, four occupational therapists received initial training in the standardized administration and scoring of the MABC-2, as well as additional consultation and guidance throughout data collection. Inter-rater agreement among the four assessors was excellent for the total score (ICC = .98).

## Outcome measures

### Resilience: Adolescent Resilience Questionnaire (ARQ)[60]

This self-report questionnaire is designed to assess personal and environmental factors underlying resilience among adolescents aged 11–19 years. Adolescents rated 88 items measuring resilience factors in five different domains (individual, family, peers, school, and community) based on their frequency, using a scale from 1 (“almost never”) to 5 (“almost always”). The total score on the questionnaire is the mean of scores across all items, ranging from 1 to 5, with higher scores indicating higher levels of resilience. The questionnaire has been found to be valid and reliable, demonstrating good psychometric properties [60,61].

Significant correlations were identified between the ARQ subscales. The strongest correlations were observed between overall resilience and two subscales: “peers” and “self” (p < .01). Notably, the peer’s subscale showed strong positive correlations with all research variables (r > .50).

### SOC: The Sense of Coherence Scale-Short Version (SOC-13) [17]

This self-report questionnaire is designed to measure SOC across its three dimensions (comprehensibility, manageability, and meaningfulness); it is intended for use across diverse populations. Adolescents rate 13 items using a scale ranging from 1 (“never”) to 7 (“always”). The total score on the questionnaire is the mean of scores across all items, ranging from 1 to 7, with a higher score indicating a higher level of SOC [17]. The questionnaire has been found to be valid and reliable, demonstrating good psychometric properties [62,63].

### Hope: The Children’s Hope Scale (CHS) [14]

This self-report questionnaire is designed to measure hope among children aged 8–16; it is intended for use across diverse populations. Adolescents rated six items measuring hope in two subscales (“agency” and “pathway”), using a scale ranging from 1 (“never”) to 6 (“always”). The total score on the questionnaire is the mean of scores across all items, ranging from 1 to 6, with a higher score indicating a higher level of hope [14]. The questionnaire has been found to be valid and reliable, demonstrating good psychometric properties [16,31,64,65]. Consistent with accepted norms in the academic literature, this study also utilized the questionnaire for ages 16–18 [19].

### Procedure

Upon receiving ethical approval from the University Institutional Review Board (Ref. #25122022), advertisements were disseminated via social media, inviting adolescents and their parents/guardians to participate in the study. Eligibility was assessed according to the criteria described in the Participants section. Recruitment took place between 26 January 2023 and 2 December 2023. A preliminary phone conversation was conducted to provide detailed information about the study and its objectives, following which written informed consent was obtained from both the adolescents and one of their guardians. Guardians provided demographic and health-related information about the adolescents and completed the ADHD-RS and DCDQ through an online survey platform. Adolescents aged 16 years and older independently completed the AAC-Q on the same platform. Reported motor-related functional difficulties were assessed using age-appropriate validated questionnaires. Parents of participants within the validated age range of the DCDQ completed the DCDQ. For older adolescents, the AAC-Q was administered because the DCDQ was not validated for their age range and the AAC-Q was specifically developed and validated for adolescents and adults [24]. Both questionnaires assessed motor-related difficulties in everyday functioning, although they differed in informant and item content. After the initial screening for ADHD and/or DCD, the MABC-2 was administered to adolescents identified as having one or both conditions. Subsequently, they also completed the ARQ, CHS, and SOC-13 questionnaires using the online survey platform.

### Statistical analysis

Statistical analysis was conducted using IBM SPSS statistical software (Version 26; IBM Corp., Armonk, NY), and statistical significance was set at *p* < .05. Descriptive statistics were employed to characterize the demographic information. For the final analysis, adolescents with ADHD-only and DCD-only were combined into a single-diagnosis ADHD/DCD group. This grouping was aligned with the central aim of the study, which was to compare adolescents with a single neurodevelopmental diagnosis, adolescents with co-occurring ADHD+DCD, and TD adolescents. Prior to combining these groups, independent-samples t-tests were conducted to examine differences between the ADHD-only and DCD-only groups in the main study variables. Homogeneity of variance was assessed using Levene’s test. Differences between groups on demographic characteristics were analyzed using Chi-square and one way Analysis of Variance (ANOVA), as appropriate. Additionally, ANOVA was conducted to assess differences between groups in the total scores of resilience, hope, and SOC; while a Multivariate Analysis of Variance (MANOVA) was conducted to examine differences between groups in the subscales (domains) of the ARQ and CHS.

A preliminary analysis using the Shapiro-Wilk test was performed to assess normality by calculating skewness and kurtosis. The variables in the study were normally distributed; thus, Pearson correlation analyses were conducted to examine the associations between resilience, hope, and SOC. Finally, stepwise hierarchal regression was conducted to assess the relationship and percentage of variance explained resilience, according to the type of diagnosis (based on group), hope, and SOC. Effect sizes were reported using partial eta squared (ηp²) for omnibus ANOVA/MANOVA effects and Hedges’ g for significant pairwise comparisons, given the unequal group sizes. Hedges’ *g* values are reported as absolute values, with the direction of group differences described in the text.

## Results

No significant differences were found among the three study groups regarding age (F (2,101) = 1.09, *p* = .34, ηp² = .02), gender (Fisher-Freeman-Halton exact test (*p* = .053), living area (Fisher-Freeman-Halton exact test *p* = .13) and educational setting (Fisher-Freeman-Halton exact test *p* = .81). Among adolescents with ADHD, ADHD-related medication did not differ significantly between the ADHD group and the ADHD+DCD group, χ²(1) = 0.00, *p* = .99. An exploratory MANOVA was conducted among participants with ADHD-only or ADHD+DCD (*N* = 51) to examine whether reported ADHD medication use was associated with resilience, hope, and SOC while accounting for diagnostic subgroup. Medication status was not significantly associated with the combined outcomes, Pillai’s trace = .010, *F*(3, 46) = 0.15, *p* = .928, ηp² = .010. Follow-up univariate tests similarly showed no significant association between medication status and resilience, *F*(1, 48) = 0.13, *p* = .717, ηp² = .003; hope, *F*(1, 48) = 0.01, *p* = .918, ηp² < .001; or SOC, *F*(1, 48) = 0.004, *p* = .950, ηp² < .001. See Table 1 for the socio-demographic characteristics of the participants.

Preliminary independent-samples *t*-tests indicated no statistically significant differences between the ADHD-only and DCD-only groups in resilience total score, t(35) = 0.59, *p* = .56, 95% CI [−0.26, 0.48]; hope, t(35) = 1.02, *p* = .31, 95% CI [−1.66, 5.04]; or SOC, t(35) = 0.17, *p* = .87, 95% CI [−0.66, 0.78]. Levene’s tests were non-significant for all three variables, indicating no significant violation of the homogeneity of variance assumption: resilience, *F* = 1.33, *p* = .26; hope, *F* = 0.74, *p* = .40; and SOC, *F* = 0.94, *p* = .34. Therefore, the ADHD-only and DCD-only groups were combined into a single-diagnosis ADHD/DCD group for the main analysis.

Regarding the overall ARQ score, Levene’s test indicated that the assumption of homogeneity of variance was met for resilience, *F*(2, 102) = 0.06, *p* = .939. A one-way ANOVA revealed a significant group difference in resilience, *F*(2, 102) = 4.01, *p* = .021, ηp² = .073. Follow-up post-hoc analysis with Bonferroni correction revealed the ADHD+DCD group reported significantly lower resilience than the TD group, *p* = .017, Hedges’ *g* = 0.72, with no other significant pairwise differences.

Regarding the ARQ subscales, Box’s M test was non-significant, Box’s *M* = 40.57, *F*(30, 18418.53) = 1.25, *p* = .166, indicating that the assumption of equality of covariance matrices was met. The MANOVA revealed a significant multivariate effect of group on the ARQ subscales, Pillai’s trace = .272, *F*(10, 198) = 3.12, *p* = .001, ηp² = .136. After false discovery rate (FDR) correction for the follow-up univariate tests, only the ARQ self-subscale remained significant, *F*(2, 102) = 9.94, *p* < .001, ηp² = .163. Levene’s test for the ARQ self-subscale was non-significant, *F*(2, 102) = 0.75, *p* = .47, indicating that the homogeneity of variance assumption was met. Follow-up post-hoc analysis with Bonferroni correction indicated that ARQ “self” subscale scores were significantly lower in both the ADHD+DCD group (*p* < .001, Hedges’ *g* = 1.12) and the single-diagnosis ADHD/DCD group (*p* = .037, Hedges’ *g* = 0.58), compared with the TD group.

Similarly, Levene’s test indicated that the assumption of homogeneity of variance was met for hope, *F*(2, 102) = 2.15, *p* = .122. A significant group difference was found in hope using one-way ANOVA, *F*(2, 102) = 6.91, *p* = .002, ηp² = .119 Follow-up post-hoc analysis with Bonferroni correction revealed that the ADHD+DCD group reported significantly lower hope scores than the TD group, *p* = .001, Hedges’ *g* = 0.87; regarding the CHS subscales, Box’s M test was non-significant, Box’s M = 11.47, *F*(6, 67738.11) = 1.85, *p* = .085. The MANOVA revealed a significant multivariate group differences, Pillai’s trace = .125, *F*(4, 204) = 3.41, *p* = .010, ηp² = .063. Levene’s tests were non-significant for both agency and pathway. After FDR correction for follow-up univariate tests, both agency, *F*(2, 102) = 7.02, FDR-adjusted *p* = .002, ηp² = .121, and pathway, *F*(2, 102) = 4.44, FDR-adjusted *p* = .014, ηp² = .080, remained significant. Bonferroni-adjusted post hoc comparisons showed that the ADHD+DCD group reported significantly lower agency scores, *p* = .001, Hedges’ *g* = 0.87, and pathway scores *p* = .011, Hedges’ *g* = 0.71 than the TD group.

In addition, regarding the SOC, Levene’s test indicated that the assumption of homogeneity of variance was met for SOC, *F*(2, 102) = 0.32, *p* = .73. A significant group difference was found in SOC, *F*(2, 102) = 5.46, *p* = .006, ηp² = .097. Post-hoc analysis indicated significant differences between the TD and co-occurrence groups in the overall SOC score, *p* = .007, Hedges’ *g* = 0.79. See Table 2.

**Table 2:** Mean scores (SD) and differences between study groups in resilience, SOC, and hope.

	ADHD/DCD	ADHDּ + DCD	TD
	n = 37	n = 23	n = 45
	M(SD) CI 95%	M(SD) CI 95%	M(SD) CI 95%
Resilience (ARQ)			
resilience total	2.46 (.47) [2.31-2.61]	2.21(.48) [2.02-2.41]	2.55 (.45) [2.41-2.69]
self	2.3 (.45) [2.15-2.44]	2.05 (.49) [1.87-2.24]	2.54 (.34) [2.41-2.67]
family	2.84 (.65) [2.61-3.07]	2.60 (.83) [2.31-2.90]	2.87 (.70) [2.66-3.09]
peers	2.88 (.59) [2.68-3.07]	2.55 (.61) [2.30-2.80]	2.78 (.60) [2.60-2.96]
school	2.28 (.69) [2.08-2.51]	2.17 (.54) [1.90-2.45]	2.40 (.69) [2.20-2.59]
community	2.23 (.85) [1.93-2.52]	1.96 (.86) [1.59-2.34]	2.06 (.99) [1.79-2.33]
SOC (SOC-13)	4.13 (.91) [3.83-4.43]	3.88(.88) [3.50-4.26]	4.57 (.82) [4.32-4.81]
hope total (CHS)	4.16 (.0.72) [3.88-4.44]	3.61(.89) [3.25-3.97]	4.43 (.95) [4.17-4.68]
agency	12.59 (2.24) [11.69-13.50]	10.87 (2.70) [9.72-12.02]	13.53 (3.17) [12.71-14.35]
pathway	12.35 (2.50) [11.44-13.27]	10.78 (2.86) [9.62-11.94]	12.91 (2.99) [12.08-13.74]

Regarding the correlation analysis conducted across all study groups combined, involving the ARQ, CHS, and SOC-13 scales, all the domains had significant correlation (*p* < .01), most of it ranging from moderate to strong (0.30–0.93). A strong positive correlation was identified between overall resilience scores, and both hope and SOC (*p* < .01), as well as between overall resilience scores and all ARQ subscales. This indicates that higher resilience scores are associated with higher levels of hope, SOC, and resilience subscale scores. See Table 3.

**Table 3:** Pearson correlations in resilience, SOC, and hope.

	Hope total	hope agency	hope pathway	ARQ total	ARQ community	ARQ school	ARQ peers	ARQ family	ARQ individual
SOC	.55**	.52**	.48**	.64**	.40**	.40**	.55**	.50**	.64**
Hope total		.93**	.91**	.66**	.35**	.60**	.52**	.41**	.60**
hope agency			.71**	.59**	.28**	.55**	.49**	.36**	.53**
hope pathway				.62**	.36**	.56**	.46**	.37**	.60**
ARQ total					.65**	.78**	.88**	.75**	.88**
ARQ community						.44**	.60**	.54**	.42**
ARQ school							.59**	.48**	.57**
ARQ peers								.82**	.63**
ARQ family									.57**

*Notes. p* < .01

Finally, to examine which of the research variables (hope, SOC, diagnosis type by group) explained the variance in the overall resilience score, a hierarchical regression analysis was conducted. In the first step, the variable for type of diagnosis (ADHD/DCD or ADHD+DCD) was entered, and the explained variance was *R*² = .073, *p* < .05. In the second step, hope and SOC variables were entered, and the explained variance increased to *R*² = .475, *p* < .001. The final model explained 54.7% of the variance in resilience, *R*² = .547. Collinearity diagnostics indicated no evidence of multicollinearity, with tolerance values ranging from .661 to .806 and VIF values ranging from 1.24 to 1.51. The type of diagnosis significantly explained resilience in the first step; however, its contribution was no longer significant after hope and SOC were added to the model. In the final model, both hope and SOC were independently and positively associated with resilience, with comparable standardized coefficients, explaining substantial variance beyond diagnostic group membership. See Table 4.

**Table 4:** Regression analysis for predicting resilience (total ARQ score).

	variables	B	SE B	β	R^2^	VIF	Δ R^2^
first step	type of diagnosis (ADHD/DCD)	−0.095	0.103	−.095	*.073	1.18	*.073
	type of diagnosis (ADHD+ DCD)	−0.337	0.119	*−.293		1.18	
second step	type of diagnosis (ADHD/DCD)	0.062	0.075	.062.	**.547	1.24	**.475
	type of diagnosis (ADHD+ DCD)	−0.001	0.09	.000		1.36	
	Hope	0.227	0.044	**.431		1.51	
	SOC	0.218	0.043	**.412		1.48	

*Notes.* *p < .05 **p < .001

## Discussion

The present study aimed to compare resilience, hope, and SOC among adolescents with ADHD/DCD, ADHD+DCD, and TD adolescents. To the best of our knowledge, this preliminary study is the first to examine these variables across these populations. Our findings demonstrated moderate to strong correlations between all study variables (resilience, hope, and SOC). These results align with existing literature, reporting positive associations between SOC and hope, although the magnitude of these associations has varied across samples and cultural groups [44,66]. Previous studies have also reported positive associations between SOC and resilience [13] and between resilience and hope [10–12]. These findings reinforce the theoretical connections between resilience, hope, and SOC, further validating their selection for this research.

Hope, SOC, and resilience have been extensively studied both independently and within the same theoretical framework [67]. On the one hand, these concepts are all linked to an individual’s ability to overcome difficulties, and thus they share similarities in their theoretical structure [14, 67]. On the other hand, hope, and SOC are distinct constructs with unique contributions. For instance, hope was found to explain 48% of the variance in resilience among adolescents from low socioeconomic backgrounds, highlighting its significant role in fostering resilience [68]; while SOC was identified as the strongest predictor and explainer of psychological distress, even more than resilience [67]. These results emphasized the unique role of each variable. Their moderate to strong correlations in the current study are consistent with the theoretical overlap among them. Nevertheless, both hope and SOC remained independently associated with resilience after accounting for diagnostic group. The addition of hope and SOC explained a further 47.4% of the variance in resilience, with the final model explaining 54.7% of the variance. This pattern suggests that, despite their shared conceptual and empirical overlap with resilience, hope and SOC each provide unique explanatory information. The substantial shared variance among resilience, hope, and SOC should be interpreted cautiously. All three constructs were assessed concurrently using self-report questionnaires and are conceptually related positive psychological constructs. Therefore, their associations may partly reflect common-method variance and conceptual overlap, in addition to meaningful relationships among the constructs. Accordingly, the regression findings indicate statistical associations rather than clinical or causal prediction.

Our primary research aim addressed group differences in the research variables. The only significant differences found were between the ADHD+DCD group and the TD group across all study variables. These results differ from prior studies that suggested lower resilience, hope, and SOC among individuals with ADHD/DCD alone, compared to TD populations [26,30,43]. However, they are consistent with studies conducted among populations with co-occurring ADHD+DCD in other variables.

Similar to the current study, Missiuna et al [69] found significant differences in clinical depressive symptoms between the ADHD+DCD and TD children, but they found no differences between the co-occurrence group and the ADHD-only or DCD-only groups. Likewise, in the study by Dewey and Volkovinskaia [49], significant differences were found between the ADHD+DCD group and TD adolescents in quality of life and bullying variables, but no differences were found between the co-occurrence group and the ADHD-only group.

These findings may be attributed to the fact that previous studies examining ADHD and DCD populations separately did not account for the possibility of co-occurrence, even though such overlap between these diagnoses is common [32]. For example, adolescents with ADHD were often included in studies without being assessed for DCD by using standard tools, and vice versa [26,43,70].

The differences we observed in the ADHD+DCD group, compared to the TD group, highlight the perception that co-occurrence presents a distinct profile, differing from each diagnosis individually [34,71]. Co-occurrence involves more brain regions and more severe symptoms [34,71–74], which can make it more challenging for adolescents with co-occurrence to develop resilience, hope, and SOC. This was further demonstrated in a study comparing coping strategies among individuals with only ADHD or DCD to those with co-occurrence, which found that individuals with co-occurrence used broader but less diverse coping strategies than those with a single diagnosis [73].

In addition, a significant difference was found between the co-occurrence and TD groups only on the “self” subscale of the ARQ. This subscale reflects areas such as self-confidence, emotional awareness, negative thinking, social skills, and empathy [60]. Tal-Saban and Kirby [75] found that young adults with co-occurring ADHD and DCD exhibited lower empathy, compared to those with DCD alone. Additionally, a lower self-concept was reported in boys with co-occurrence of ADHD and DCD, compared to those with DCD alone [76]. These studies may help explain the differences between the groups in the “self” subscale.

However, no significant differences were found between the study groups in the other ARQ subscales (“family,” “peers,” “school,” and “neighborhood”). These subscales are recognized as key resilience factors linked to better outcomes for young people facing adversity [60]. The absence of differences may be explained by the similarity in living areas and educational settings across our study groups. Since the ARQ assesses resilience in various ecological contexts [60], it is possible that shared environmental factors- such as school policies, community resources, and family support structures-contributed to these similarities.

In the current study, significant differences were found in hope and its subscales, as well as in SOC, between the co-occurrence group and the TD group. SOC is a health-related construct influenced by the burden of symptoms [77], while hope is a resource for health and health-promoting processes [15]. Thus, higher symptom severity is often associated with lower levels of hope and SOC [29,78]. These findings help explain our results, in that the emotional profile of the co-occurrence group appeared to be more severe than that of the other groups, as the burden of symptoms in the co-occurrence group is likely higher than in the ADHD/DCD group [34].

Surprisingly, no differences were found between the ADHD/DCD group and the TD groups in study variables exempt the “self” subscale in the ARQ. This finding is broadly consistent with previous evidence of lower self-esteem and greater social and emotional difficulties among young people with ADHD or DCD [79–81]. A possible explanation for the lack of differences in other variables such as resilience is the ability of these adolescents to develop effective coping strategies. Khairati et al [82], found that adolescents with DCD, despite reporting challenges, emphasized their resilience and the importance of recognizing their strengths and developing coping strategies. Similarly, research suggests that individuals with ADHD possess certain strengths, such as high energy and enhanced focus, which may support resilience development [83,84]. Studies comparing adolescents with and without ADHD have found no significant differences in resilience and strengths [85,86], while Chan et al [87] reported that children with ADHD demonstrated resilience similar to that of their TD peers.

The absence of significant differences between the single-diagnosis and TD groups was inconsistent with some previous findings. Earlier studies reported lower levels of SOC and hope in children with DCD, compared to their TD peers [31], and lower levels of resilience in adults with DCD and suspected DCD compared to their TD peers [30]. However, it is important to note that those studies included both children and adults, and may not have accounted for ADHD diagnoses, which could have influenced the results. Additionally, the small sample size of the DCD sub group in the present study may may also have reduced the ability to detect differences.

The current study has several limitations. First, ADHD classification was based only on a parent-reported physician diagnosis together with a parent-report ADHD symptom scale. Information regarding ADHD medication type such as dosage and treatment duration was not collected. Therefore, the potential role of medication in resilience, hope, and SOC could not be fully examined. Second, regarding the screening questioners, the use of age-appropriate motor questionnaires resulted in a change in informant, with parents completing the DCDQ for younger adolescents and older adolescents completing the AAC-Q themselves. Although both measures assess motor-related difficulties in everyday functioning, differences between parent and self-report may have introduced measurement variability across age groups. Third, the MABC-2 was not administered to the TD group, limiting certainty regarding the absence of performance-based motor difficulties in that group. In addition, the MABC-2 was administered to participants aged 17–18 years, beyond its standardized normative age range. The occupational therapists administering MABC-2 were not blinded to participants’ screening status or study group. Although standardized procedures and predefined cutoffs were used, assessor expectancy bias cannot be fully excluded.

Regarding the outcome measures, resilience, hope, and SOC were assessed concurrently using self-report measures; therefore, conceptual overlap and shared-method variance may have increased the observed associations. Furthermore, in this study, the sample was relatively small and unequal across groups. This imbalance limited statistical power and the precision of group comparisons and may partly reflect the under recognition of DCD. Future studies should replicate these findings in larger, more balanced, and better-characterized samples while incorporating longitudinal and mixed-method design to provide a more comprehensive understanding of adolescents’ positive adaptation resources and coping experiences. Moreover, future studies should examine resilience, hope, and SOC across a broader range of NDDs.

## Conclusion

To our knowledge, this is one of the first studies to examine differences in resilience, hope, and SOC among adolescents with ADHD/DCD, co-occurring ADHD+DCD, and TD peers. Preliminary findings suggest that adolescents with co-occurring ADHD and DCD may report lower resilience, hope, and SOC than TD peers. The findings highlight the importance of considering positive adaptation resources in adolescents with these NDDs. Accordingly, these resources may be considered alongside motor, attentional, and functional difficulties during assessment and may inform individualized, strengths-based support involving adolescents, families, educators, and clinicians. Given the preliminary nature of the study, the findings should be interpreted cautiously and replicated in larger, more balanced samples.
